# Vitamin C modulates the levels of several proteins of the mitochondrial complex III and its activity in the mouse liver

**DOI:** 10.1016/j.redox.2022.102491

**Published:** 2022-09-24

**Authors:** Lucie Aumailley, Sylvie Bourassa, Clarisse Gotti, Arnaud Droit, Michel Lebel

**Affiliations:** a Centre de recherche du CHU de Québec, Faculty of Medicine, Université Laval, Québec City, Québec, G1V 4G2, Canada; b Proteomics Platform, Centre de recherche du CHU de Québec, Faculty of Medicine, Université Laval, Québec City, Québec, G1V 4G2, Canada

**Keywords:** Vitamin C, Gulonolactone oxidase, Mass spectrometry, Mitochondrial complex III, Reactive oxygen species, Sex-related differences, Liver, Mouse

## Abstract

Ascorbate is a crucial antioxidant and essential cofactor of biosynthetic and regulatory enzymes. Unlike humans, mice can synthesize ascorbate thanks to the key enzyme gulonolactone oxidase (Gulo). In the present study, we used the *Gulo*^*−/−*^ mouse model, which cannot synthesize their own ascorbate to determine the impact of this vitamin on the liver proteome of specific subcellular organelles. We performed label-free Liquid Chromatography-Tandem Mass Spectrometry (LC-MS/MS) global quantitative proteomic profiling to identify and quantify proteins in microsomal enriched liver extracts (MEE) from *Gulo*^*−/−*^ mice treated with 0–0.4% (w/v) ascorbate in drinking water until the age of four months. Using a principal component analysis on normalized and imputed data of the label-free protein quantifications, a sex-based difference in MEE proteome profiles was observed for all the different ascorbate treated mice. Suboptimal hepatic ascorbate concentrations affected the levels of more proteins and hence biochemical processes in females than in males. Nevertheless, Pearson correlation analyses revealed that the MS intensities of various proteins involved in complement activation inversely correlated with liver ascorbate concentrations in both *Gulo*^*−/−*^ males and females. Moreover, the correlation analyses also indicated that several proteins in the mitochondrial complex III of the electron transport chain positively correlated with liver ascorbate concentrations in both *Gulo*^*−/−*^ females and males. Consequently, the mitochondrial complex III activity in *Gulo*^*−/−*^ female and male mice treated with suboptimal hepatic concentrations of ascorbate was significantly lower than *Gulo*^*−/−*^ mice treated with optimal ascorbate concentration. Finally, the whole liver of ascorbate-deficient *Gulo*^*−/−*^ mice exhibited lower ATP levels and increased reactive oxygen species. These findings provide new information on how ascorbate deficiency potentially induces mitochondrial dysfunction in the liver of mice.

## Graphical abstract

**Figure:**
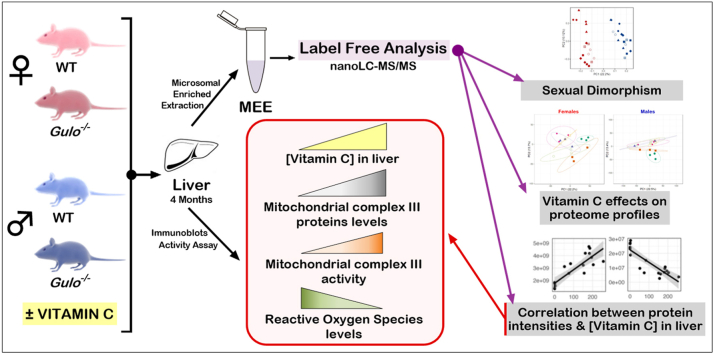

## 1 Introduction

In addition to its well-known ability to scavenge and neutralize free radicals, vitamin C (ascorbate) is an important cofactor of biosynthetic and regulatory enzymes including hydroxylases, dioxygenases, and histone or DNA demethylases. Such enzymes affect collagen and neurotransmitters synthesis, epigenetic regulation, as well as the function of the immune system [1,2]. Unable to produce their own ascorbate, humans entirely rely on dietary intake to prevent vitamin C deficiency. According to epidemiological studies, hypovitaminosis C, or low levels of vitamin C, is a common feature among subpopulations (between 5% and 30%) depending on socioeconomic status, smoking habit, and age [[3], [4], [5], [6]]. Hypovitaminosis C is also becoming a pressing issue in the elderly and in chronically ill hospitalized patients [[7], [8], [9], [10]]. Importantly, low blood levels of ascorbate have been inversely related to several chronic diseases and their associated risk factors, including C-reactive protein [11], blood pressure [12], diabetes [13], metabolic syndrome [14,15], cardiovascular diseases [16], and all-cause mortality [17].

Mice are particularly useful for thoroughly investigating the interplay of environmental factors, including dietary supplementations, and physiologic pathways in the pathogenesis of multifactorial diseases. However, unlike humans, mice are able to synthesize ascorbate thanks to the key enzyme gulonolactone oxidase (Gulo) [18]. Consequently, mouse tissues generally have sufficient levels of ascorbate such that the impact of exogenous ascorbate supplementations can be difficult to interpret in experimental settings. In this context, the *Gulo*^*−/−*^ mouse is a relevant model that depends entirely on ascorbate derived from the diet [19]. Importantly, the levels of ascorbate found in the serum of *Gulo*^*−/−*^ mice reflect the amounts of ascorbate provided in drinking water and can be controlled in a non-invasive manner [20,21]. A previous targeted mass spectrometry study on male *Gulo*^*−/−*^ mice supplemented with various ascorbate concentrations in their drinking water showed important alterations in their serum metabolic profiles [20]. Mainly, the ratio of serum arginine/lysine, tyrosine/phenylalanine, and specific species of saturated/unsaturated lipids changed in male *Gulo*^*−/−*^ mice depending on the amount of ascorbate in drinking water [20]. Recently, a global quantitative proteomic approach on the serum of both male and female *Gulo*^*−/−*^ mice supplemented with various ascorbate concentrations revealed that the serum proteome profiles differed significantly between male and female mice [21]. Nonetheless, serum proteins involved in retinoid metabolism, cholesterol, and lipid transport were similarly affected by serum ascorbate levels in both male and female *Gulo*^*−/−*^ mice [21]. Importantly, these proteins are secreted in the blood by the liver.

The liver is an organ that plays a pivotal role in nutrient, drug, hormone, cytokine, and metabolic waste product processing. Hepatocytes are the chief functional cells of the liver representing roughly 80% of the liver's mass and secrete a large number of proteins in the blood [22]. Interestingly, ascorbate deficiency in *Gulo*^*−/−*^ mice has been associated with swollen hepatic mitochondria. In addition, the density of hepatic mitochondria envelop area was decreased among *Gulo*^*−/−*^ mice treated with low ascorbate concentrations compared to wild type littermates [20]. Given the role of hepatic mitochondria in oxidative phosphorylation and lipid metabolism, ascorbate supplementations and consequently ascorbate body levels in *Gulo*^*−/−*^ mice may directly impact on such essential mitochondrial functions. Moreover, in male *Gulo*^*−/−*^ mice, levels of hepatic phosphorylated endoplasmic reticulum associated stress markers IRE1α and eIF2α correlated inversely with serum ascorbate levels suggesting that ascorbate not only modulates endoplasmic reticulum stress response but also plays a key role in the healthy functioning of this organelle [20]. Note that stressed and ultimately dysfunctional subcellular organelles like the endoplasmic reticulum or the mitochondria in the hepatocytes can lead to major impairments of many metabolic and detoxification hepatic activities. However, the precise impacts of how low levels of ascorbate on various biological processes in the liver may lead to a disease-prone status are still unclear. Identifying proteins involved in physiologic/metabolic pathways that are affected by ascorbate may help unveil the effects of this vitamin on those pathways in the liver and contributes to the increased understanding of its role in prevention and progression of metabolic diseases.

In the present study, we used label-free Liquid Chromatography-Tandem Mass Spectrometry (LC-MS/MS) global quantitative proteomic profiling to identify proteins in the hepatic tissues that correlate with the levels of liver ascorbate in *Gulo^−/−^* mice treated with different concentrations of ascorbate in drinking water. Since we previously reported that ascorbate deficient *Gulo^−/−^* males exhibited stresses in the endoplasmic reticulum and the mitochondria of their liver [20], we performed quantitative proteomic profiling on microsomal enriched liver extracts from our different ascorbate treated cohorts of *Gulo^−/−^* males and females. This subcellular enrichment step allowed us to focus on proteins from the mitochondrial and endoplasmic reticulum organelles. We found that the MS intensities of several proteins in the mitochondrial complex III of the electron transport chain correlated positively with liver ascorbate concentrations in both *Gulo^−/−^* males and females. In contrast, proteins involved in complement activation correlated negatively with liver ascorbate concentrations in both *Gulo^−/−^* males and females. Finally, ascorbate-deficient *Gulo^−/−^* mice exhibited decreased mitochondrial complex III activities, lower ATP levels, and increased reactive oxygen species (ROS) in their liver tissue.

## 2 Materials and methods

### 2.1 Animals and maintenance

*Gulo^−/−^* mice were obtained from the Mutant Mouse Regional Resource Centers (University of California Davis, CA) and were housed at the Centre Hospitalier de l’Université Laval animal facility and maintained with 0.4% (w/v) of l-ascorbate (vitamin C; Sigma-Aldrich, Oakville, ON) in drinking water. These mice were backcrossed onto the C57BL/6NHsd background (Harlan Laboratories, Frederick, MD) for 12 generations. Finally, heterozygous mice were crossed to obtain *Gulo^−/−^* and wild type (*Gulo^+/+^*) mice. This study was carried out in strict accordance with the recommendations in the Guide for the Care and Use of Laboratory Animals of the Canadian Council on Animal Care in science and the protocol was approved by the Committee on the Ethics and Protection of Animal of Laval University (Permit Number: CHU-18-036). Mice were housed in cages (containing a top filter) at 22 ± 2 °C with 40%–50% humidity and a 12-h light–dark cycle (light cycle: 07:00–19:00 h). All mice were fed ad libitum with Teklad Global 18% protein rodent diet, 6% fat, 110 IU/kg of vitamin E, and 15 IU/g of vitamin A (Envigo cat. # 2918, Madison, WI).

**Fig. 1:**
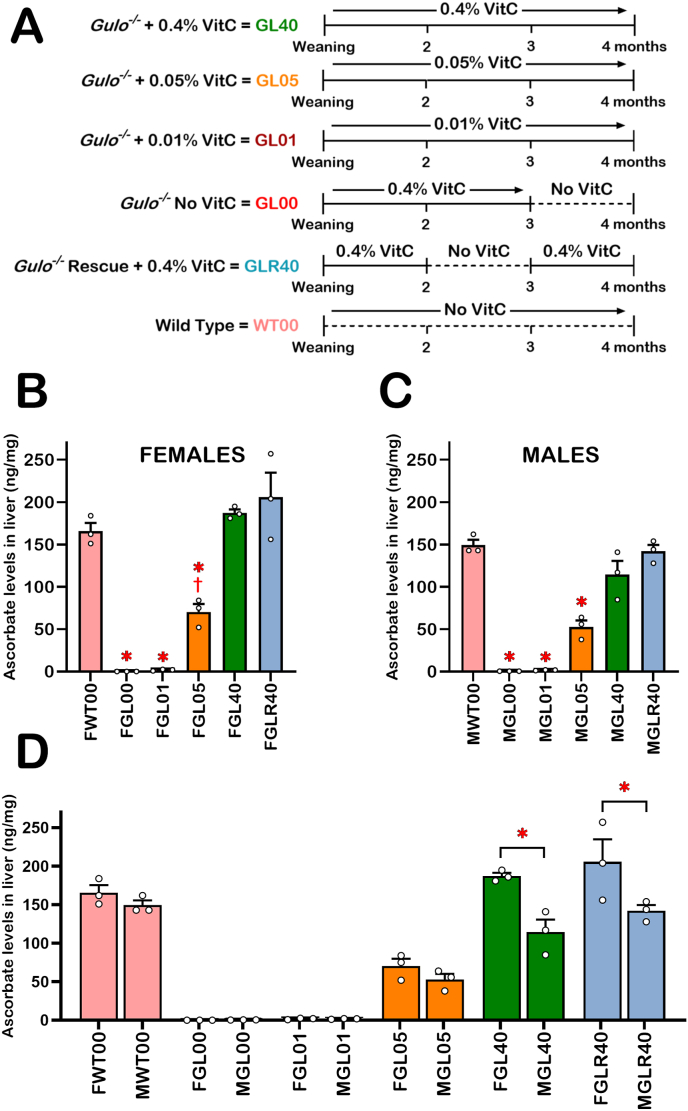
Depiction of the experimental design and levels of ascorbate in the liver of mice. (A) Males and females were separated into six experimental groups. Each group contained three males and three females. Animals were labeled according to their genotype (GL for *Gulo*^*−/−*^ mice and WT for wild-type mice) and the vitamin C (VitC) treatments (% is weight of ascorbate per 100 mL of drinking water). The vitamin C (or ascorbate) treatment sessions for each group are depicted on the top of each timeline. The liver was harvested at the age of 4 months for all mice. (B) Histogram showing the ascorbate levels in the liver of each indicated female cohorts (N = 3 females per group). * = significantly different compared to FWT00, FGL40, and FGLR40 (with a Tukey post ANOVA tests *p*-value < 0.05). † = significantly different compared to FGL00 and FGL01 (with a Tukey post ANOVA tests *p*-value < 0.05). (C) Histogram showing the ascorbate levels in the liver of each indicated male cohorts (N = 3 males per group). * = significantly different compared to MWT00, MGL40, and MGLR40 (with a Tukey post ANOVA tests *p*-value < 0.05). (D) Histogram comparing liver ascorbate levels between the different groups of males and females receiving the same ascorbate treatments. Bars in each histogram represent mean ± SEM. Significant differences are indicated using * (with a Tukey post ANOVA tests *p*-value < 0.05).

Mice were separated into six cohorts containing three males and three females each (see Fig. 1A for summary). One cohort of *Gulo^−/−^* mice was maintained on standard diet and supplemented with 0.4% ascorbate (w/v) in drinking water from weaning until the age of four months (referred as GL40). A second cohort of *Gulo^−/−^* mice was treated with 0.05% ascorbate in drinking water from weaning until the age of four months (GL05). A third cohort of *Gulo^−/−^* mice was supplemented with 0.01% ascorbate from weaning until the age of four months (GL01). A fourth cohort of *Gulo^−/−^* mice was treated with 0.4% ascorbate until the age of three months. Ascorbate was then removed from drinking water for four weeks (GL00). Mice were not kept beyond four weeks without ascorbate in drinking water as they were losing more than 15% of their body weight and became moribund [20]. A fifth cohort of *Gulo^−/−^* mice were treated with 0.4% ascorbate from weaning until the age of two months. Ascorbate was removed from drinking water for one month. Then ascorbate was added back (0.4%) to drinking water until the mice reached the age of four months (GLR40). This cohort was considered the vitamin C rescue cohort. Finally, wild type control (*Gulo^+/+^*) mice were maintained in the same room with no ascorbate supplementation in drinking water and were used as our normal reference cohort (WT00) (Fig. 1A).

### 2.2 Liver collection from the different mouse cohorts

Mice were fasted overnight before liver collection. Liver was collected at 10:00 a.m. the next day after blood harvesting by cardiac puncture and final exsanguination under general anesthesia (with 3% isoflurane) at the age of four months. Liver samples were frozen at −80 °C until microsomal enriched extraction and further analyses.

### 2.3 Microsomal enriched extraction from mouse livers

**Fig. 2:**
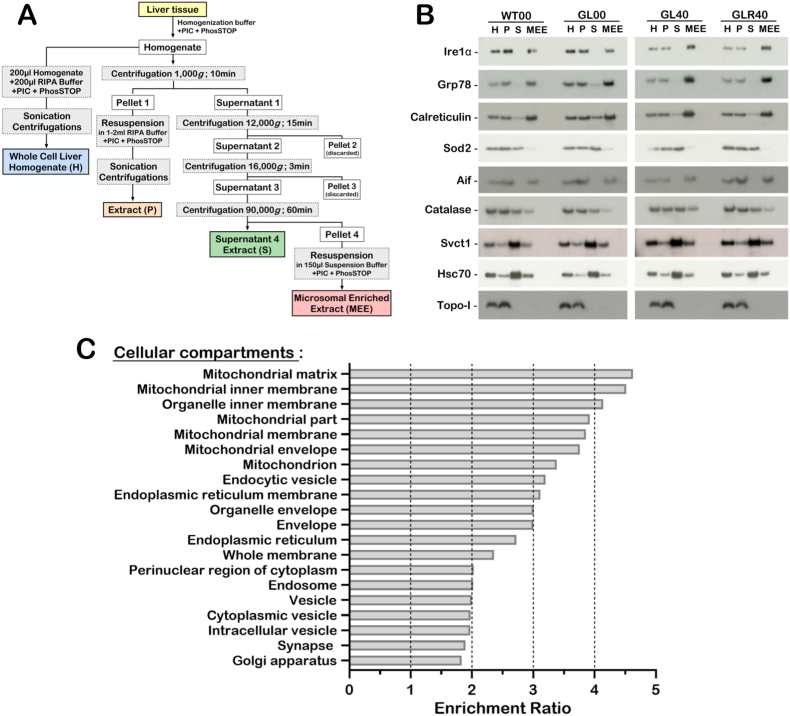
Reproducibility of the liver microsomal enrichment extraction procedure on the different groups of mice. (A) Schematic representation of the different steps undertaken to obtain different cellular fractions. (B) Example of western blots showing protein levels of IRE1α, Grp78, calreticulin, Sod2, Aif, catalase, Svct1, Hsc70, and topoisomerase I (Topo-I) in the different liver fractions. Each lane contains 15 μg of proteins. (H = whole cell homogenate; P = pellet fraction from the first step of the procedure; S = supernatant fraction of the last step of the procedure; MEE microsomal enriched extract). (C) Gene ontology analysis of the proteins identified by mass spectrometry showing the enriched cellular compartments after the MEE procedure.

Microsomal enriched extracts were obtained using an endoplasmic reticulum enrichment assay kit (Novus Biologicals, Burlington, ON) following the manufacturer's protocol with some modifications (Fig. 2A). Briefly, weighed liver tissues were homogenized in isosmotic homogenization buffer containing phosphatase inhibitor cocktail (PhosSTOP™ from Roche Applied Science, Indianapolis, IN) and protease inhibitor cocktail provided by the kit. An aliquot of the homogenate (whole cell liver fraction H in Fig. 2A) was kept for Western blot analysis. The rest of the homogenate was centrifuged at 1000 *g* for 10 min at 4 °C to obtain pellet 1 and supernatant 1. The pellet 1 was dissolved for 1 h at 4 °C in RIPA Buffer (50 mM Tris-HCl (pH 7.5), 150 mM NaCl, 1% NP-40, 0.2% SDS, 1% sodium deoxycholate, 1 mM phenylmethylsulfonylfluoride, complete protease inhibitor cocktail and phosphatase inhibitor cocktail) to obtain a final pellet extract (P in Fig. 2A) for Western blot analysis. The supernatant 1 was recentrifuged at 12,000 *g* for 15 min at 4 °C to obtain pellet 2 and supernatant 2. Pellet 2 was discarded and the supernatant 2 was briefly recentrifuged at 16,000 *g* for 3 min at 4 °C after which a new supernatant 3 was centrifuged at 90,000 *g* for 1 h at 4 °C. The final supernatant extract (S in Fig. 2A) was frozen for Western blot analysis. The final pellet obtained at the end of the procedure was finally dissolved using the suspension buffer provided by the kit with protease and phosphatase inhibitor cocktails and was referred as the microsomal enriched extract (MEE in Fig. 2A). Protein concentration was determined by the Bradford protein assay (Bio-Rad, Mississauga, ON). Samples were frozen at −80 °C until mass spectrometry and immunoblotting analyses.

### 2.4 Immunoblotting analysis and coomassie-brilliant-blue staining

Protein samples were resolved using 8, 10, or 12% sodium dodecyl sulfate-polyacrylamide gel electrophoresis and then transferred onto 0.2 μm polyvinylidene difluoride membrane (EMD Millipore Corporation, Burlington, MA). After incubating 1 h with blocking solution (PBS-T or TBS-T containing 5% non-fat milk), the membrane was probed overnight at 4 °C with a primary antibody. After washing with PBS-T or TBS-T, species-specific horseradish peroxidase-conjugated secondary antibody was added for 2 h at room temperature (Cell Signaling Technology, Beverly, CA). Signals were generated with Immobilon® Forte Western HRP Substrate from EMD Millipore Corporation. When indicated, immunoblots were probed with the following antibodies: rabbit polyclonal antibodies raised against heat shock cognate 71 kDa protein (anti-HSP70/HSC70 (H300): sc-33575) and sodium dependent vitamin C transporter (anti-SVCT1 (H-78): sc-30113) from Santa Cruz Biotechnology (Santa Cruz, CA); rabbit monoclonal antibodies against inositol-requiring kinase 1α (anti-IRE1 (14C10) #3294) and calreticulin (anti-calreticulin (D3E6) XP® #12238) from Cell Signaling Technology (Beverly, MA); a rabbit polyclonal antibody against glucose-related protein 78 (anti-GRP78) from Proteintech™ (Chicago, IL); a rabbit monoclonal antibody against topoisomerase 1 (anti-topoisomerase I [EPR5375] ab109374) from Abcam (Cambridge, MA); a rabbit polyclonal antibody against mitochondrial manganese superoxide dismutase (anti-MnSOD #06–984) from EMD Millipore Corporation (Temecula, CA); mouse monoclonal antibodies against catalase (C0979) and β-actin (A5441) from Sigma-Aldrich (Oakville, ON) and mouse monoclonal antibodies against ubiquinol-cytochrome C reductase core protein I (anti-Uqcrc1 [16D10AD9AH5] ab110252), ubiquinol-cytochrome C reductase core protein II (anti-Uqcrc2 [13G12AF12BB11] ab14745), ubiquinol-cytochrome c reductase, Rieske iron-sulfur polypeptide 1 (anti-Uqcrfs1 [5A5] ab14746), NADH dehydrogenase [ubiquinone] 1 β subcomplex subunit 8 (anti-Ndufβ8 [20E9DH10C12] ab110242), Succinate dehydrogenase [ubiquinone] iron-sulfur subunit (anti-Sdhb [21A11AE7] ab14714), and ATP synthase subunit α (anti-ATP5α [15H4C4] ab14748). To evaluate total liver protein status in our different samples, proteins were resolved using 12% sodium dodecyl sulfate-polyacrylamide gel electrophoresis. The proteins in the gels were stained and visualized with a Coomassie-brilliant-blue staining solution (12.5 mg of Coomassie Brilliant blue R 250 (C.I. 42660) from Sigma-Aldrich was dissolved into 100 mL of a solution containing 40% methanol and 5.3% glacial acetic acid).

### 2.5 Sample preparation for vitamin C (ascorbate) quantification in whole cell liver samples

All solutions were prepared with ultrapure water containing 0.5% metaphosphoric acid. Ascorbic acid, ascorbic acid-^13^C_6_ and metaphosphoric acid were obtained from Sigma Aldrich (St-Louis, MO). Formic acid, methanol and acetonitrile of LC-MS grade were purchased from Fisher Scientific (Pittsburgh, PA). Liver tissues (approximately 100 mg) were homogenized with the soft tissue CK14 lysis kit from VWR (Radnor, PA) in a Precellys 24 tissue homogenizer (Bertin Instruments, France) with 1 mL of ice-cold metaphosphoric 0.5% solution. Samples were then centrifuged at 21,380 *g* for 10 min at 4 °C. The supernatants were then passed through a Sep-Pak cartridge CN and a 0.2 μm GHP Acrodisc filter (Waters, Milford, MA). Filtered samples were diluted appropriately and ascorbic acid-^13^C_6_ solution was added to all samples as an internal standard before analysis.

### 2.6 Ascorbic acid LC-MS/MS analysis in whole cell liver samples

Analyses were performed using an ACQUITY™ UPLC system coupled with an ACQUITY™ triple quadrupole tandem mass spectrometer Xevo-TQD (Waters, Milford, MA), operating in multiple reaction monitoring (MRM) and negative electrospray ionization (ESI) modes. Ascorbic acid and ascorbic acid-^13^C_6_ were chromatographically separated using a Synergi Polar-RP column (2.0 × 100 mm, 2.5 μm particle size) supplied by Phenomenex (Torrance, CA) at 40 °C. The mobile phases used were solvent A (water 0.1% formic acid) and solvent B (acetonitrile 0.1% formic acid) under linear gradient elution conditions at a flow rate of 0.4 mL/min. The gradient started at 100% of A for 4 min, reaching 100% of B at 5 min, maintaining 100% B for 1 min, reaching 100% A at 6.5 min, and stabilizing the column for 5.5 min for an optimized gradient of 12 min. Samples and standards injection volume was 5 μL. The MS parameters at the optimized conditions were as follow: capillary 1.5 kV, desolvatation temperature 600 °C, desolvatation flow 1000 L/h. Transitions of the analyte and internal standard were as follow: 175.0 > 87.0 and 175.0 > 115.0, cone voltage 30 V and collision energy 10 V for ascorbic acid and 181.0 > 90.0, cone voltage 20 V and collision energy 10 V for ascorbic acid-^13^C_6_. All data were acquired using Masslynx™ software (Waters, Milford, MA).

### 2.7 Sample preparations for label-free Liquid Chromatography-Tandem Mass Spectrometry

Sodium deoxycholate (DOC) and pepstatin were added to the microsomal enriched extracts to obtain final concentrations of 1% and 1 μM, respectively. Sample homogenization was performed by sonication on ice with microprobe (SonicDismembrator 550, Fisher Scientific) 20 times (1 s on/1 s off), and then centrifuged at 16,000 *g* for 15 min. Supernatant was precipitated with 5 vol of acetone overnight. The protein pellet was recovered by centrifugation at 16,000 *g* for 15 min and resuspended in 200 μL of 1% DOC/50 mM ammonium bicarbonate buffer. A Bradford protein assay was performed to estimate protein concentration.

Ten μg of proteins from the MEE were digested with trypsin. Briefly, proteins were first reduced with 0.2 mM dithiothreitol (DTT) for 30 min at 37 °C and alkylated with 0.8 mM iodoacetamide for 30 min at 37 °C. Samples were then incubated with trypsin (trypsin:protein; 1:50) at 37 °C overnight. The reaction was stopped by addition of 1% trifluoroacetic acid (TFA), 0.5% acetic acid, and 0.5% acetonitrile then centrifuged for 5 min at 16,000 *g*. The peptides obtained were then desalted using C18 stagetip.

### 2.8 Label-free liquid chromatography-tandem mass spectrometry analysis of MEE samples

One μg of each MEE sample was analyzed by nanoLC/MSMS using a Dionex UltiMate 3000 nanoRSLC chromatography system (Thermo Fisher Scientific, San Jose, CA) connected to an Orbitrap Fusion mass spectrometer (Thermo Fisher Scientific) equipped with a nanoelectrospray ion source. Peptides were trapped at 20 μL/min in loading solvent (2% acetonitrile, 0.05% TFA) on a 5 mm × 300 μm C18 pepmap cartridge pre-column (Thermo Fisher Scientific) for 5 min. Then, the pre-column was switched online with Pepmap Acclaim column (Thermo Fisher Scientific) 50 cm × 75 μm internal diameter separation column and the peptides were eluted with a linear gradient from 5 to 40% solvent B (A: 0,1% formic acid, B: 80% acetonitrile, 0.1% formic acid) in 90 min, at 300 nL/min for a total run time of 120 min. Mass spectra were acquired using a data dependent acquisition mode using Thermo XCalibur software version 4.1.50. Full scan mass spectra (350–1800 *m*/*z*) were acquired in the orbitrap using an AGC target of 4e5, a maximum injection time of 50 ms, and a resolution of 120,000. Internal calibration using lock mass on the *m*/*z* 445.12003 siloxane ion was used. Each MS scan was followed by acquisition of fragmentation MS/MS spectra of the most intense ions for a total cycle time of 3 s (top speed mode). The selected ions were isolated using the quadrupole analyzer in a window of 1.6 *m/z* and fragmented by Higher energy Collision-induced Dissociation (HCD) with 35% of collision energy. The resulting fragments were detected by the linear ion trap in rapid scan rate with an AGC target of 1e4 and a maximum injection time of 50 ms. Dynamic exclusion of previously fragmented peptides was set for a period of 30 s and a tolerance of 10 ppm.

### 2.9 Database searching and Label Free Quantification (LFQ)

Spectra were searched against the Uniprot Ref *Mus musculus* database (July 2020 release/63807 entries) using the Andromeda module of MaxQuant software v. 1.6.10.43 [23]. Trypsin/P enzyme parameter was selected with two possible missed cleavages. Carbamidomethylation of cysteins was set as fixed modification while methionine oxidation, protein N-terminal acetylation and hydroxyproline were set as variable modifications for the global search. Mass search tolerance were 5 ppm and 0.5 Da for MS and MS/MS, respectively. For protein validation, a maximum False Discovery Rate of 1% at peptide and protein level was used based on a target/decoy search. MaxQuant was also used for Label Free Quantification. The ‘match between runs’ option was used with 20 min value as alignment time window and 0.7 min as match time window. Only unique and razor peptides were used for quantification. Normalisation (LFQ intensities) was performed by MaxQuant.

### 2.10 LFQ data availability

All mass spectrometry data (raw files and MaxQuant search result files) are publicly available on ProteomeXchange repository (www.proteomexchange.org) with the identifier PXD035312.

### 2.11 LFQ data post-processing and statistical analysis

RStudio 1.2.5019 was used for data post-processing. Some protein intensity values were missing, there were replaced by a noise value corresponding to 1% percentile of the normalized value for each condition. A protein was considered as quantifiable only if at least three intensity values in the three replicates of one of the two conditions being compared were present and if two peptides or more were identified for this protein. A ratio of LFQ intensity means, a Z-score, and a limma *q*-value (Benjamin-Hochberg correction for multiple testing) were calculated. To be significantly differentially expressed, a protein needed to have a *q*-value less than 0.05 and a Z-score lower than −1.96 (under-expressed) or higher than 1.96 (over-expressed).

Principal Component Analysis (PCA) was performed with the MixOmics R package or ClustVis [24].

One-way ANOVA followed by Tukey's honest significant difference test were performed for ascorbate concentrations using GraphPad Prism version 9.3.1. Differences were considered significant at a *p*-value < 0.05.

Pearson correlation coefficients (*r*) were calculated for each protein between the LFQ intensities as function of the liver ascorbate concentrations (ng/mg of tissue). A correlation between the protein abundance and the ascorbate concentration was considered as significant if the Pearson correlation coefficient *r* > |0.5897| (for N = 18, *p*-value = 0.01). Only proteins with a Pearson correlation coefficient *r* > |0.5897| (for N = 18, *p*-value = 0.01) and an absolute ratio between GL40/GL00 group >2 were considered for the analysis. Finally, proteins missing LFQ intensity values in more than five samples were excluded from the analysis.

### 2.12 Measurement of mitochondrial complex III activity

MEE samples were obtained as described above. Twenty μg of MEE from female and male samples from each GL00, GL01, and GL40 group were used to quantify complex III activity. Mitochondrial complex III activity (Mitochondrial Complex III Activity Assay Kit, #MAK360, Sigma-Aldrich, Oakville, ON) in MEE samples were measured according to the manufacturer's instructions. Briefly, the reduction of cytochrome *c* through the activity of complex III was measured at 550 nm after 30 min of reaction. Absorbance was measured with a SpectraMax® i3 multi-mode microplate reader (Molecular Devices, LLC., San Jose, CA). To evaluate the net complex III activities in MEE samples, antimycin A (an inhibitor of the mitochondrial complex III) was added in one well as indicated by the kit and the net complex III activities were calculated as followed: Net complex III activity in sample = activity in reaction without antimycin A – activity in reaction with antimycin A were used for the assay.

### 2.13 Measurement of liver adenosine triphosphate (ATP) levels

For ATP levels measurement, liver tissues from both females and males of each GL00, GL01, and GL40 group were used. ATP levels were determined in the whole liver using a luminescent assay kit (#ab65313 from Abcam, Cambridge, MA) following the manufacturer's protocol. Approximately 60 μg of liver tissue was homogenized in 100 μL of the nucleotide releasing buffer provided by the kit. The homogenates were incubated on ice for 10 min and centrifuged at 16,000 *g* for 15 min at 4 °C. For the assay, 100 μL prepared reaction mix, containing the ATP converting enzyme, was added in wells and the background luminescence was read (Data A), then 20 μL of whole liver lysates was added per well. After 2 min, the luminescence was read (Data B). The ATP levels of each liver lysates were calculated as followed: Data B – Data A. Results were expressed as units of luminescence per μg of proteins. The luminescence was measured with a SpectraMax® i3 multi-mode microplate reader (Molecular Devices, LLC., San Jose, CA).

### 2.14 Reactive oxygen species (ROS) measurements in whole liver tissues

Whole liver tissues from females and males of each GL00, GL01, and GL40 group were lysed in RIPA buffer. Approximately 500 μg of protein lysates were incubated with 10 μg/mL of the dye 2′-7′ dichlorofluorescein diacetate (Sigma-Aldrich, Oakville, ON) for 1h at 37 °C into 96-well black plate with clear bottom. Upon oxidation, this dye is highly fluorescent. The excitation and emission wavelengths used were 485 and 527 nm, respectively. Background fluorescence, measured at the beginning of the assay, were extracted from the dichlorofluorescein value recorded after 1 h of reaction, for each sample. Results were expressed as units of fluorescence per μg of proteins. Fluorescence was measured with a SpectraMax® i3 multi-mode microplate reader (Molecular Devices, LLC., San Jose, CA).

## 3 Results

### 3.1 Experimental groups of mice and vitamin C (ascorbate) levels

To gain information on the biological processes that are altered in the microsomal enriched extracts of mice exhibiting different degrees of vitamin C deficiency, we first set out to study specific cohorts of female and male mice treated with various concentrations of ascorbate in drinking water. Fig. 1A shows the different mouse lines (*Gulo*^*−/−*^ and WT mice) treated with the indicated concentrations of ascorbate in drinking water. Briefly, six different cohorts containing three males and three females were used in this study. *Gulo^−/−^* mice were treated from weaning until the age of four months with 0.4% (GL40), 0.05% (GL05) or 0.01% (GL01) ascorbate (w/v). One cohort of *Gulo^−/−^* mice underwent a four-week ascorbate depletion from the age of three months until the age of four months (GL00). Another cohort of *Gulo^−/−^* mice were supplemented with 0.4% ascorbate (w/v) from weaning to the age of two months after which they experienced a four-week ascorbate depletion. At the age of three months, 0.4% ascorbate (w/v) was added back to the drinking water until the age of four months (GLR40). Finally, wild type control mice (WT00), which can produce their own ascorbate, were used in this study as normal reference cohort without ascorbate supplementation in drinking water.

Since the levels of ascorbate found in the serum of *Gulo*^*−/−*^ mice reflected the amounts of ascorbate provided in drinking water [20,21], we examined the amount of ascorbate in the whole liver tissue to see if there was any correlation between serum and liver ascorbate levels. Using UPLC-MS/MS, the amount of ascorbate stored in the whole liver tissue of mice was measured for each treatment group (Fig. 1A). Comparisons between groups of mice showing significant differences (one-way ANOVA on all female and male groups followed by Tukey's multiple comparisons tests) are highlighted in the supplementary Table S1. As indicated in Fig. 1B (female groups) and 1C (male groups), minimal ascorbate could be detected in the liver of ascorbate-depleted *Gulo^−/−^* mice (FGL00 and MGL00). The treatment of female and male *Gulo^−/−^* mice with 0.01% ascorbate (FGL01 and MGL01) since weaning did not increase hepatic ascorbate levels significantly compared to FGL00 and MGL00 mice. The treatment of female *Gulo^−/−^* mice with 0.05% ascorbate (FGL05) significantly increased the hepatic ascorbate level compared to the levels observed with 0.01% ascorbate (FGL01) or when ascorbate was depleted (FGL00) (Fig. 1B; † *p*-value <0.05). An increase of the hepatic ascorbate level was also observed in male *Gulo^−/−^* mice treated with 0.05% ascorbate (MGL05), but this increase was not significantly higher than MGL00 or MGL01 (Fig. 1C). In contrast, the ascorbate levels measured in the liver of untreated wild type (FWT00 and MWT00) as well as *Gulo^−/−^* mice treated with 0.4% ascorbate since weaning or for four weeks (FGL40, MGL40, FGLR40 and MGLR40) were significantly higher than all the *Gulo^−/−^* mice treated with suboptimal levels of ascorbate in drinking water (FGL00, MGL00, FGL01, MGL01, FGL05, and MGL05) (Fig. 1B and C; * *p*-value <0.05).

**Fig. S1:**
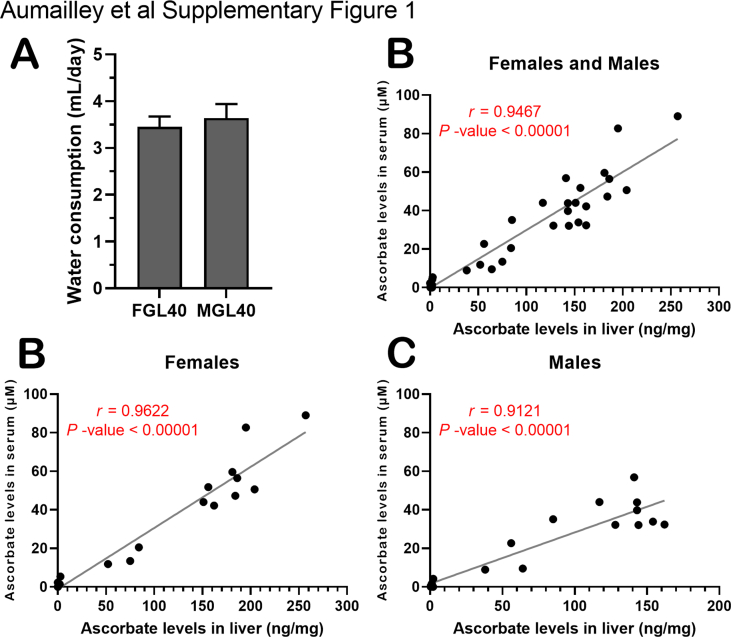
Correlation of liver ascorbate and serum ascorbate in our mouse cohorts. (A) Histogram representing daily water consumption in Gulo^−/−^ females (FGL40) and males (MGL40) treated with 0.4% of ascorbate (w/v) in drinking water at three months of age. Bars in the histogram represent SEM (N = 7 females and 10 males). (B) Correlation of liver ascorbate and serum ascorbate in females and males (N = 36). (C) Correlation of liver ascorbate and serum ascorbate in females (N = 18). (D) Correlation of liver ascorbate and serum ascorbate in males (N = 18).

Interestingly, when we compared the ascorbate levels in females and males experiencing the same ascorbate treatments, only the *Gulo^−/−^* females treated with 0.4% ascorbate in drinking water since weaning (FGL40) or for four weeks (rescue experiment; FGLR40) exhibited significantly more liver ascorbate levels than their male counterparts upon similar treatments (MGL40 and MGLR40) (Fig. 1D; * *p*-value <0.05). We examined the daily water consumption of *Gulo^−/−^* mice treated with 0.4% ascorbate in drinking water since weaning. There was no significant difference in overall daily water consumption between females (FGL40) and males (MGL40) indicating that these *Gulo^−/−^* mice were exposed to the same amount of ascorbate (Fig. S1A).

Finally, since the ascorbate levels in serum of the exact same mice had already been measured [21], we determined whether there was a correlation between the ascorbate levels in serum and liver (Table S2). When we analyzed both males and females together (N = 36), we found that the ascorbate levels in liver correlated significantly with the ascorbate levels in serum with a Pearson's correlation coefficient *r* of 0.9467 (*p*-value <0.00001) (Fig. S1B). When we separated the animals according to their sex and performed the same analysis (N = 18), we also obtained a significant positive correlation between ascorbate levels in liver and serum in both females and males (Figs. S1C and D).

### 3.2 Reproducibility of the microsomal enriched extraction procedure across different mouse groups

Physiological anomalies have been reported in cellular organelles such as mitochondria and the endoplasmic reticulum in the liver of ascorbate-deficient *Gulo^−/−^* mice [20]. To unveil the biochemical pathways affected by ascorbate in such organelles, we performed proteomic profiling of microsomal enriched extracts to identify the proteins changing with ascorbate levels in the liver. Prior to in-depth proteomic analysis, we first estimated the efficiency of our microsomal enriched extraction procedure by monitoring the levels of nine specific subcellular protein markers using Western blot analyses in different groups of mice experiencing various degrees of vitamin C deficiency. A schematic of the microsomal enriched extraction procedure is presented in Fig. 2A. Different fractions were collected and included the whole cell liver homogenate (H), the first pellet extract (P), the supernatant 4 extract of the last centrifugation (S), and the microsomal enriched extract (MEE) (Fig. 2A). We validated the reproducibility of this procedure on wild type mice (WT00), ascorbate-depleted *Gulo^−/−^* mice (GL00), 0.4% ascorbate treated *Gulo^−/−^* mice (GL40), and *Gulo^−/−^* mice from the rescue experiment (GLR40) (Fig. 1A). Representative immunoblots of the analyzed proteins are shown in Fig. 2B for one animal of each group of mice. The endoplasmic reticulum markers Ire1α, Grp78, and calreticulin proteins were mainly detected in the microsomal enriched extract (MEE) after the whole homogenate (H) and pellet (P) fractions were processed. The mitochondrial Sod2 proteins gave a weak signal in the MEE samples. In contrast, the mitochondrial Aif protein was detected in the MEE samples but not in the supernatant 4 extract (S). The peroxisomal catalase enzyme was detected in all the examined fractions. The cell surface membrane ascorbate transporter Svct1 gave a weak signal in the MEE samples but a strong signal in the supernatant 4 extract (S). The Hsc70 protein gave the most intense signal in the supernatant 4 extract (S) as it is mainly a cytoplasmic protein. Finally, topoisomerase I was not detected in the MEE samples and the supernatant 4 extract (S). These results indicate that our microsomal enriched extracts contained proteins from subcellular organelles like the endoplasmic reticulum, mitochondria, and peroxisomes. Importantly, an ascorbate deficiency in mice (GL00) did not impact on the enrichment procedure compared to mice treated with optimal levels of ascorbate (GL40 and GLR40).

### 3.3 Microsomal proteome profiles of *Gulo*^*-/-*^ mice treated with optimal levels of ascorbate compared to wild type (*Gulo^+/+^*) reference mice

**Fig. 3:**
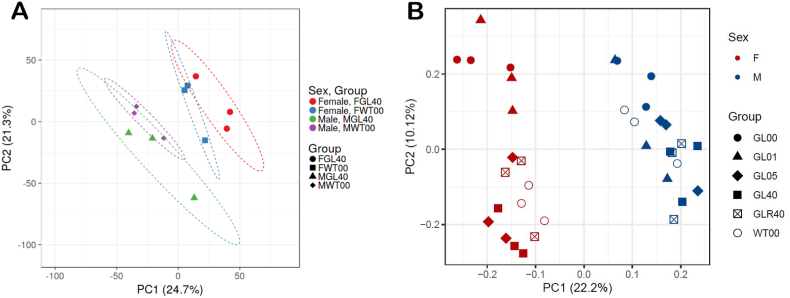
Impact of sexual dimorphism on the MEE proteome profiles of mice treated with different concentrations of ascorbate. (A) PCA graph of *Gulo*^*−/−*^ mice treated with 0.4% ascorbate in drinking water since weaning compared to age-matched control untreated WT mice in both females and males. (B) PCA graph of *Gulo*^*−/−*^ mice treated with different concentrations of ascorbate in drinking water demonstrating the difference between males and females. F = Females and M = males; WT00 = WT females or males with no ascorbate for 4 months; WT40 = WT females or males treated with 0.4% ascorbate for 4 months; GL00 = *Gulo*^*−/−*^ females or males with no ascorbate for 1 month; GL01 = *Gulo*^*−/−*^ females or males treated with 0.01% ascorbate for 4 months; GL05 = *Gulo*^*−/−*^ females or males treated with 0.05% ascorbate for 4 months; GL40 = *Gulo*^*−/−*^ females or males treated with 0.4% ascorbate for 4 months; GLR40 = *Gulo*^*−/−*^ females or males treated with 0% ascorbate for 1 month followed by 1 month of 0.4% ascorbate treatment.

To determine whether the microsomal proteomic profiles differ between *Gulo^−/−^* mice treated with optimal levels of ascorbate (0.4% in drinking water) and wild type mice, we performed quantitative proteomic profiling with label-free LC-MS/MS. Three males and three females of each cohort constituting biological replicates were used for label-free quantification analyses. A total of 3276 proteins in the MEE samples were identified (Table S3). Gene ontology analysis using the WEB-based GEne SeT AnaLysis Toolkit (http://www.webgestalt.org) indicated that the MEE identified proteins were enriched for mitochondrial, endoplasmic reticulum, and cytoplasmic vesicles components (Fig. 2C). We performed a principal component analysis (PCA) using LFQ normalized and imputed data. We observed that the general MEE proteome profile of male *Gulo^−/−^* mice treated with 0.4% ascorbate overlapped with the general MEE proteome profile of male wild type mice (Fig. 3A). Similarly, the general MEE proteome profile of female *Gulo^−/−^* mice treated with 0.4% ascorbate showed overlaps with the general MEE proteome profile of female wild type mice. Interestingly, however, the MEE proteome profiles did not overlap between males and females in our PCA analysis (Fig. 3A). These results indicate that the MEE profiles of *Gulo^−/−^* mice treated with 0.4% ascorbate and thus exhibiting same levels of liver ascorbate concentrations as in wild type mice overlaps with the MEE proteome profiles of untreated wild type mice. However, a clear distinction between males and females MEE proteome profiles was perceptible among *Gulo^−/−^* mice treated with 0.4% ascorbate and their wild type littermates.

### 3.4 Sex-based difference in MEE proteome profiles

Since the MEE proteome profiles were influenced by the sex of the animals among the four different groups previously considered (Fig. 3A), we generated a PCA analysis using the proteins identified in all the mice cohorts of both sexes (12 groups, 36 animals) (Table S3). As depicted on Fig. 3B, all the female mice (*Gulo^−/−^* and wild type mice, various red symbols on the graph) clustered together and did not overlap with the male mice (*Gulo^−/−^* and wild type mice, various blue symbols on the graph). These results confirmed a sex-related difference in the MEE proteome profiles of our various WT and *Gulo^−/−^* mouse cohorts. Females and males were therefore separated into two different groups (of 18 animals each) for further analyses.

### 3.5 Liver ascorbate levels impact the hepatic MEE proteome profiles of mice

**Fig. 4:**
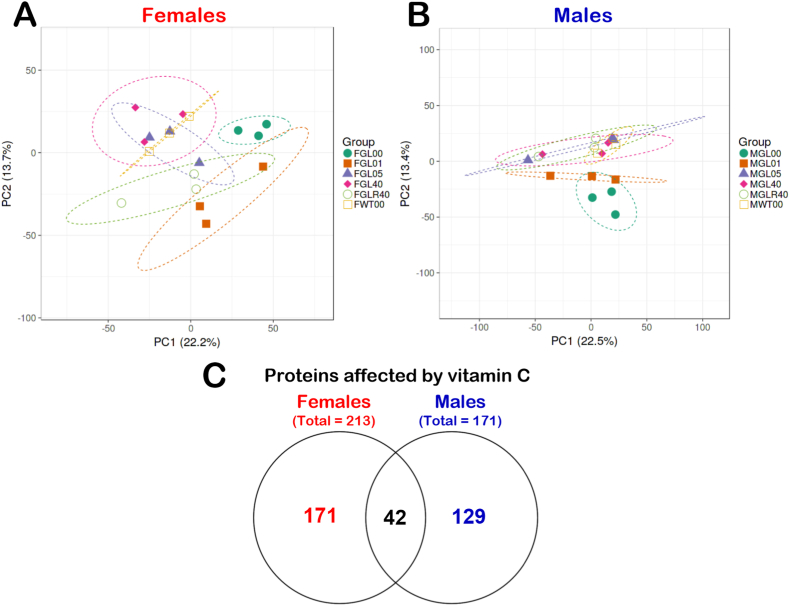
Impact of ascorbate on the MEE proteome profiles of females and male mice. (A) PCA graph of untreated WT and *Gulo*^*−/−*^ females treated with different concentrations of ascorbate in drinking water. (B) PCA graph of untreated WT and *Gulo*^*−/−*^ males treated with different concentrations of ascorbate in drinking water. The ellipses on the graphs represent the areas in which additional mice of the same group would fall based on their MEE proteome profile with a probability of 95%. (C) Venn diagram showing the number of proteins uniquely or commonly affected in male and female mice.

To determine the impact of ascorbate on the MEE proteome profiles in mice, we independently analyzed our results obtained from females and males. We first examined the general MEE proteome profile of our female cohort by generating a PCA graph (Fig. 4A) using the LFQ normalized and imputed data (Table S3). Overall, the PCA approach revealed that the three *Gulo^−/−^* females treated with no ascorbate in drinking water for four weeks (FGL00, closed green circles in Fig. 4A) clustered together and their MEE proteome profile appeared unique in comparison to all the other female groups. The *Gulo^−/−^* females treated with 0.01% ascorbate since weaning (FGL01, closed orange squares in Fig. 4A), the *Gulo^−/−^* females treated with 0.05% ascorbate since weaning (FGL05, purple triangles) and the *Gulo^−/−^* females from the rescue experiment (FGLR40, green circles) showed some overlaps on the graph. The *Gulo^−/−^* females treated with 0.05% (FGL05, purple triangles), 0.4% ascorbate since weaning (FGL40, pink lozenges), and the wild type females (FWT00, opened yellow squares) extensively overlapped. Finally, when we compared the *Gulo^−/−^* females treated with no ascorbate in drinking water for four weeks (FGL00), the *Gulo^−/−^* females treated with 0.4% ascorbate since weaning (FGL40) and the *Gulo^−/−^* females that experienced a four-week depletion followed by a four-week 0.4% vitamin C treatment (FGLR40), we observed that the MEE proteome profiles of these three groups were different and not overlapping. This result indicated that the MEE proteome profile of ascorbate-deficient *Gulo^−/−^* females was not re-established by a four-week treatment of 0.4% ascorbate in drinking water.

Similar analyses were performed for the male cohorts. As shown in Fig. 4B, the *Gulo^−/−^* males treated with no ascorbate in drinking water for four weeks (MGL00, closed green circles) clustered together near the *Gulo^−/−^* males treated with 0.01% ascorbate since weaning (MGL01, closed orange squares). The MEE proteome profiles of these two groups (MGL00 and MGL01) did not overlap with the other mice treated with higher concentrations of ascorbate or with the wild type males. The *Gulo^−/−^* males treated with 0.05% ascorbate since weaning (MGL05, purple triangles), the *Gulo^−/−^* males treated with 0.4% ascorbate since weaning (MGL40, pink lozenges), the *Gulo^−/−^* males from the rescue experiment (MGLR40, opened green circles), and the wild type males (MWT00, opened yellow squares) clustered all together. Interestingly, the *Gulo^−/−^* males that experienced a four-week ascorbate depletion followed by a four-week 0.4% ascorbate treatment (MGLR40) clustered together with the *Gulo^−/−^* males that never experienced an experimental ascorbate deficiency (MGL40). Importantly, MGL40 and MGLR40 groups did not overlap on the graph with the *Gulo^−/−^* males (MGL00) experiencing an ascorbate depletion right before harvesting their liver. These results indicate that the MEE proteome profile in ascorbate-deficient *Gulo^−/−^* males was mainly re-established by a four-week treatment of 0.4% ascorbate in drinking water to levels seen in *Gulo^−/−^* males that never experience an ascorbate deficiency in contrast to *Gulo^−/−^* females.

Out of all the proteins that could be identified in the MEE samples, 2504 proteins could be quantified between the different female cohorts (Table S4) and 2399 proteins could be quantified between the different male cohorts (Table S5). To generate two lists of proteins that showed significant differences between the six groups of females or males, we calculated the mean of the LFQ intensities for each group, the fold change, the *q*-value, and the Z-score for each two-by-two comparison. Out of all the quantified proteins in females, 213 proteins differed significantly in at least one of the comparisons between the various female groups with a *q*-value < 0.05 and a |Z-score| > 1.96 (Table S4). Out of all the quantified proteins in males, 171 proteins differed significantly in at least one of the comparisons between the various male groups with a *q*-value < 0.05 and a |Z-score| > 1.96 (Table S5). Comparison of these lists indicated that 42 proteins significantly altered by ascorbate levels were common in both females and males (Fig. 4C). Enrichments of specific biological processes that were altered by ascorbate levels in our different groups of mice (Tables S4 and S5) were evaluated using the Database for Annotation, Visualization and Integration Discovery (DAVID) tool [25]. The analysis indicated that the oxidation-reduction process (Table 1) was affected in both groups of females and males treated with different concentrations of ascorbate in drinking water. In addition to alterations in proteins involved in the electron transport from ubiquinol to cytochrome *c* in mitochondria, females also exhibited alterations in proteins involved in steroid metabolic process (Table 1).

**Table 1:** Biological processes analyses of proteins that were differentially found across the various groups of female or male MEE samples.

FEMALES		
Biological Processes	Bonferroni *p*-value	Proteins
Oxidation-reduction process	6.14E-10	Uqcrb, Sdr39u1, Plod1, Uqcr10, Mpo, Cyp3a16, Uqcrh, Cyp17a1, Adh4, Mthfd1l, Cyp2b10, Fth1, Ldhd, Phyhd1, Uqcrfs1, Hmox1, Cyc1, Scd1, Cyp2f2, Ndufv3, Cyp3a44, Cyp4b1, Cyp4a14, Coq6, Cyp39a1, Uqcrq, Cyp3a41a, Uqcrc1, Rdh5, L2hgdh, Uqcrc2, Ido2
Mitochondrial electron transport from ubiquinol to cytochrome *c*	7.79E-07	Uqcrb, Uqcrq, Uqcrc1C1, Uqcr10, Uqcrc2, Cyc1, Uqcrh
Steroid metabolic process	0.04014	Cyp39a1, Cyp2b10, Cyp3a41a, Cyp3a44, Sult2a8, Rdh5, Cyp3a16, Cyp17a1, Sult2a1

MALES		
Biological Process	Bonferroni *p*-value	Proteins
Oxidation-reduction process	9.60E-07	Cyp4a12a, Steap3, Uqcrb, Maoa, Msmo1, Plod1, Uqcr10, Higd1a, Uqcrh, Cyp2b9, Aldh1b1, Mthfd1l, Fth1, Me1, Rdh14, Scd1, Cyp2f2, Fmo2, Cyp4a14, Cyp7b1, Dhfr, Tmx1, Zadh2, Pir

### 3.6 Significant correlation between LFQ intensities of proteins from the MEE samples and liver ascorbate levels in female and male mice

**Fig. 5:**
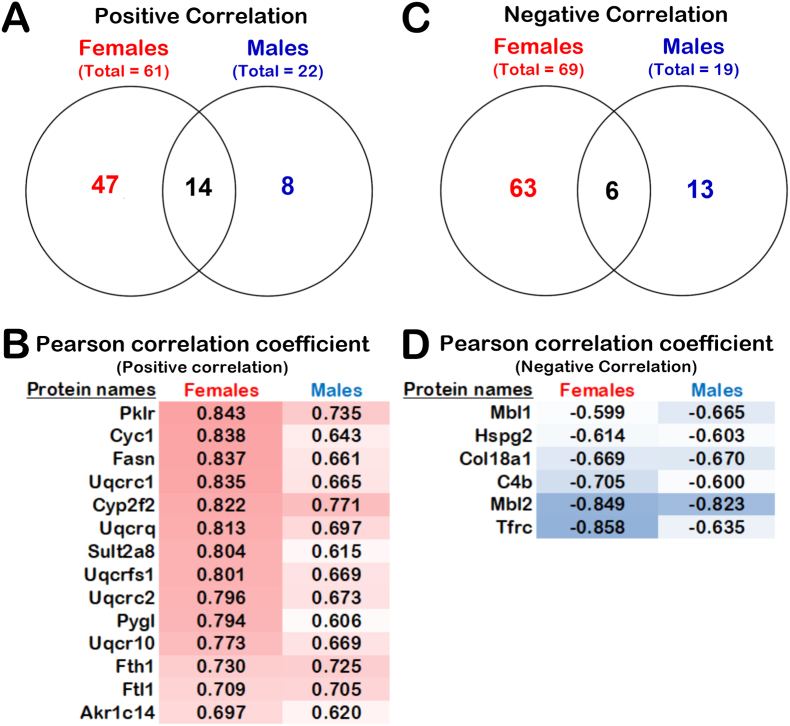
Proteins correlating with liver ascorbate levels. (A) Venn diagram showing the number of proteins uniquely or commonly correlating positively with liver ascorbate liver in male and female mice (Pearson correlation with a *p*-value < 0.01). (B) List of common proteins in female and males correlating significantly (Pearson correlation coefficient *r* > |0.5897| for N = 18; *p*-value < 0.01) and positively with liver ascorbate levels in our mouse cohorts. (C) Venn diagram showing the number of proteins uniquely or commonly correlating negatively with liver ascorbate levels in male and female mice (Pearson correlation with a *p*-value < 0.01). (D) List of common proteins in female and males correlating significantly (Pearson correlation coefficient *r* > |0.5897| for N = 18; *p*-value < 0.01) and negatively with liver ascorbate levels in our mouse cohorts.

**Fig. S2:**
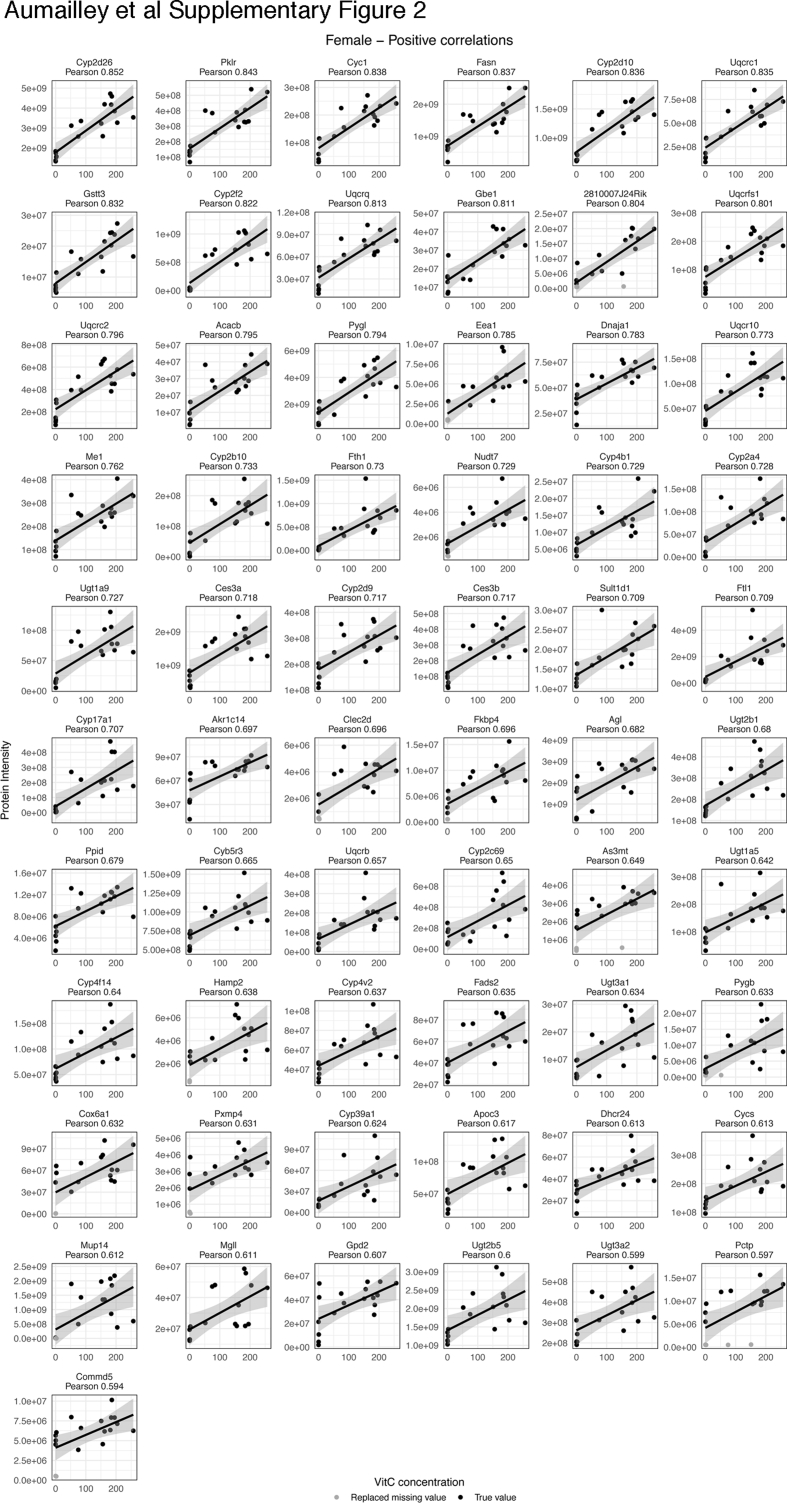
Graphs representing the significant positive correlations of quantified MEE proteins by LFQ with liver ascorbate levels (VitC concentration) in our female cohorts.

**Fig. S3:**
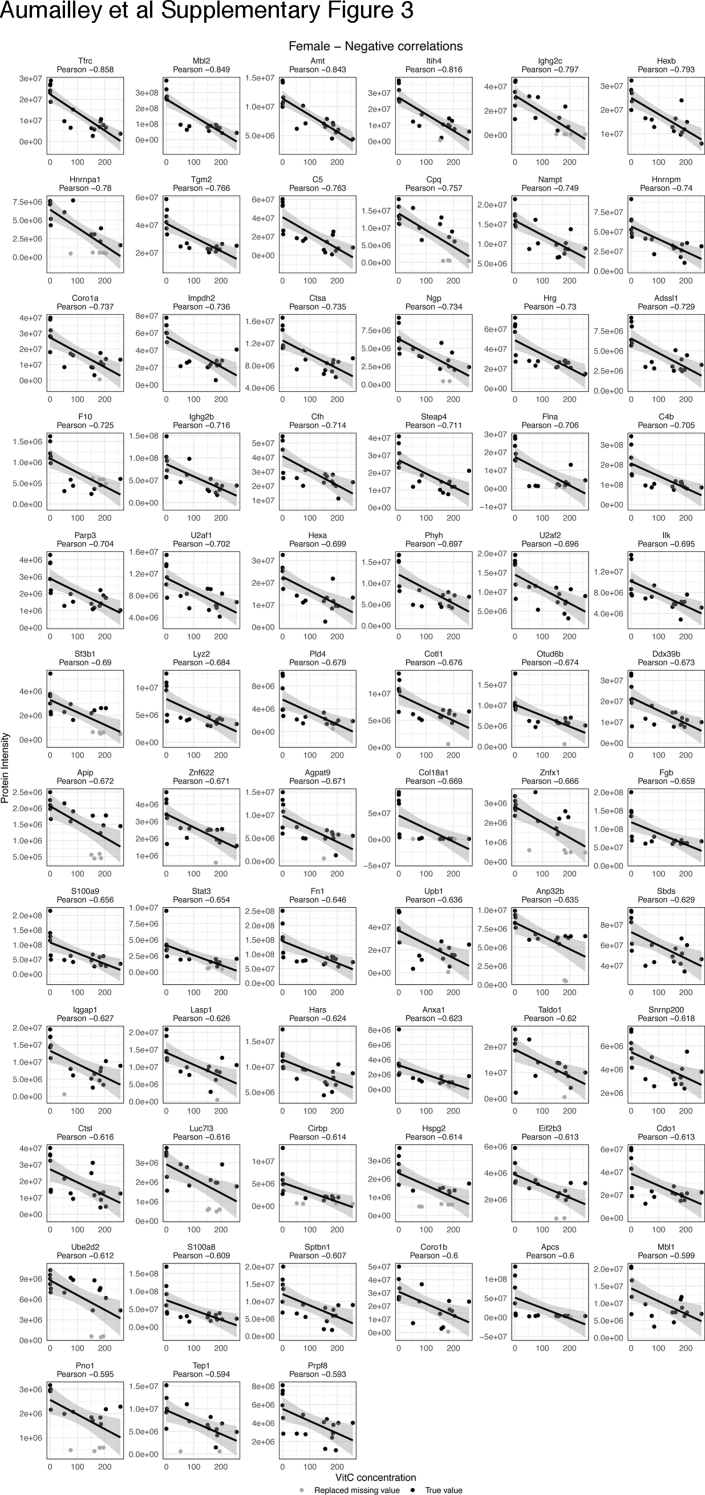
Graphs representing the significant negative correlations of quantified MEE proteins by LFQ with liver ascorbate levels (VitC concentration) in our female cohorts.

**Fig. S4:**
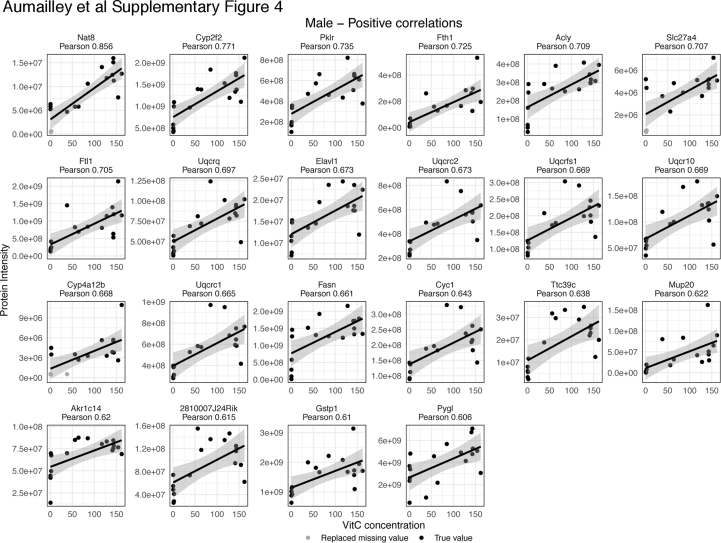
Graphs representing the significant positive correlations of quantified MEE proteins by LFQ with liver ascorbate levels (VitC concentration) in our male cohorts.

**Fig. S5:**
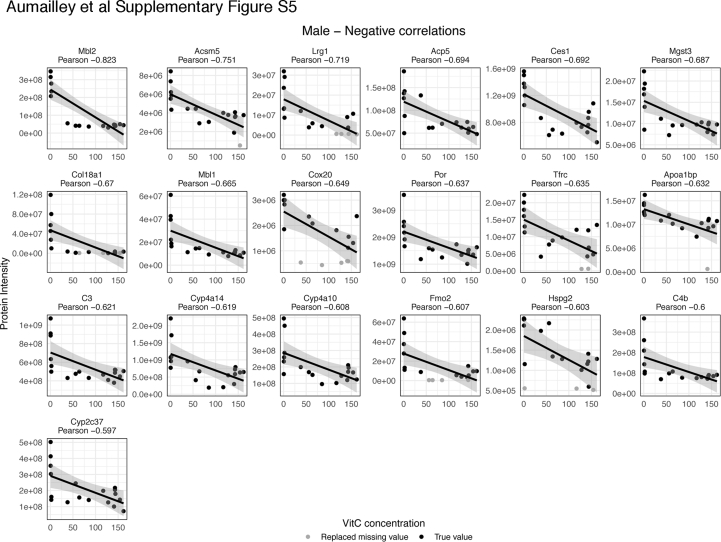
Graphs representing the significant negative correlations of quantified MEE proteins by LFQ with liver ascorbate levels (VitC concentration) in our male cohorts.

We searched in our lists of quantifiable proteins from female and male LFQ analyses, which proteins had LFQ intensities that correlated positively or negatively with liver ascorbate levels in all our mouse cohorts. Statistical associations between LFQ intensities of the MEE quantified proteins and the liver ascorbate levels were determined by Pearson correlation coefficient analyses. We focused on proteins that were significantly different with at least a two-fold change between ascorbate-deficient *Gulo^−/−^* mice and *Gulo^−/−^* mice treated with 0.4% ascorbate in drinking water since weaning. A correlation was considered significant with a Pearson coefficient *r* > |0.5897| and a *p*-value < 0.01 for N = 18 females or males. As indicated in Fig. 5A, there were 61 and 22 proteins correlating positively with liver ascorbate levels in females and in males, respectively (see Table S6 for the name of the proteins). Interestingly, 14 of these proteins were commonly found in both female and male mice (Fig. 5B). Fig. 5C shows that 69 and 19 proteins negatively correlated with liver ascorbate levels in females and in males, respectively (see Table S6 for the name of the proteins). Six of these proteins were common in both female and male mice (Fig. 5D). Figs. S2–S5 show the positive and negative correlation graphs of each of these proteins for females and males. We determined which biological processes correlated with hepatic ascorbate in females and males. Enrichments of distinct biological processes were evaluated using DAVID analysis tool [25]. Proteins that correlated positively with hepatic ascorbate levels in females are involved in mitochondrial electron transport (ubiquinol to cytochrome *c*), steroid metabolic process, lipid metabolic process, xenobiotic process, organic acid metabolic process, and the epoxygenase P450 pathway (Table 2). Proteins that correlated inversely with hepatic ascorbate levels in females are involved in RNA splicing, mRNA processing, and the innate immune response. Proteins that correlated positively with hepatic ascorbate levels in males are involved in mitochondrial electron transport (ubiquinol to cytochrome *c*) (Table 2). No specific biological process with a significant Bonferroni *p*-value was found for proteins correlating inversely with hepatic ascorbate levels in males.

**Table 2:** Gene ontology analyses of proteins that correlated with liver ascorbate levels in female or male mice.

Positive correlation in females	Bonferroni *p*-value	Proteins
Mitochondrial electron transport, ubiquinol to cytochrome *c*	1.75E-08	Uqcrb, Uqcrq, Uqcrc1, Cycs, Uqcrfs1, Uqcr10
Steroid metabolic process	8.54E-06	Cyp39a1, Cyb5r3, Cyp2b10, Akr1c14, Sult2a8, Ugt2b5, Dhcr24, Cyp17a1
Lipid metabolic process	4.07E-04	Cyp39a1, Fads2, Sult1d1, Cyb5r3, Sult2a8, Ugt2b5, Apoc3, Dhcr24, Ugt1a9, Acacb, Mgll, Cyp17a1
Xenobiotic metabolic process	0.00126	Cyp2c69, Cyp2d9, Cyp2a4, Cyp2b10, Sult2a8, Cyp2f2
Organic acid metabolic process	0.00218	Cyp2c69, Cyp2d9, Cyp2a4, Cyp2b10, Cyp2f2
Epoxygenase P450 pathway	0.02889	Cyp2c69, Cyp2a4, Cyp2b10, Cyp2f2
Inverse correlation in females	Bonferroni p-value	Proteins
RNA splicing	0.00178	Hnrnpm, Ddx39b, U2af2, Luc7l3, U2af1, Hnrnpa1, Snrnp200, Sf3b1, Prpf8
Innate immune response	0.00702	Fgb, C4b, Ighg2b, Apcs, Ighg2c, Anxa1, Cfh, Pld4, S100a9, S100a8, Mbl1, Mbl2
mRNA processing	0.01205	Hnrnpm, Ddx39b, U2af2, Luc7l3, U2af1, Hnrnpa1, Snrnp200, Sf3b1, Prpf8

Positive correlation in males	Bonferroni *p*-value	Proteins
Mitochondrial electron transport, ubiquinol to cytochrome *c*	7.74E-08	Uqcrq, Uqcrc1, Uqcrfs1, Uqcr10, Cyc1

**Fig. 6:**
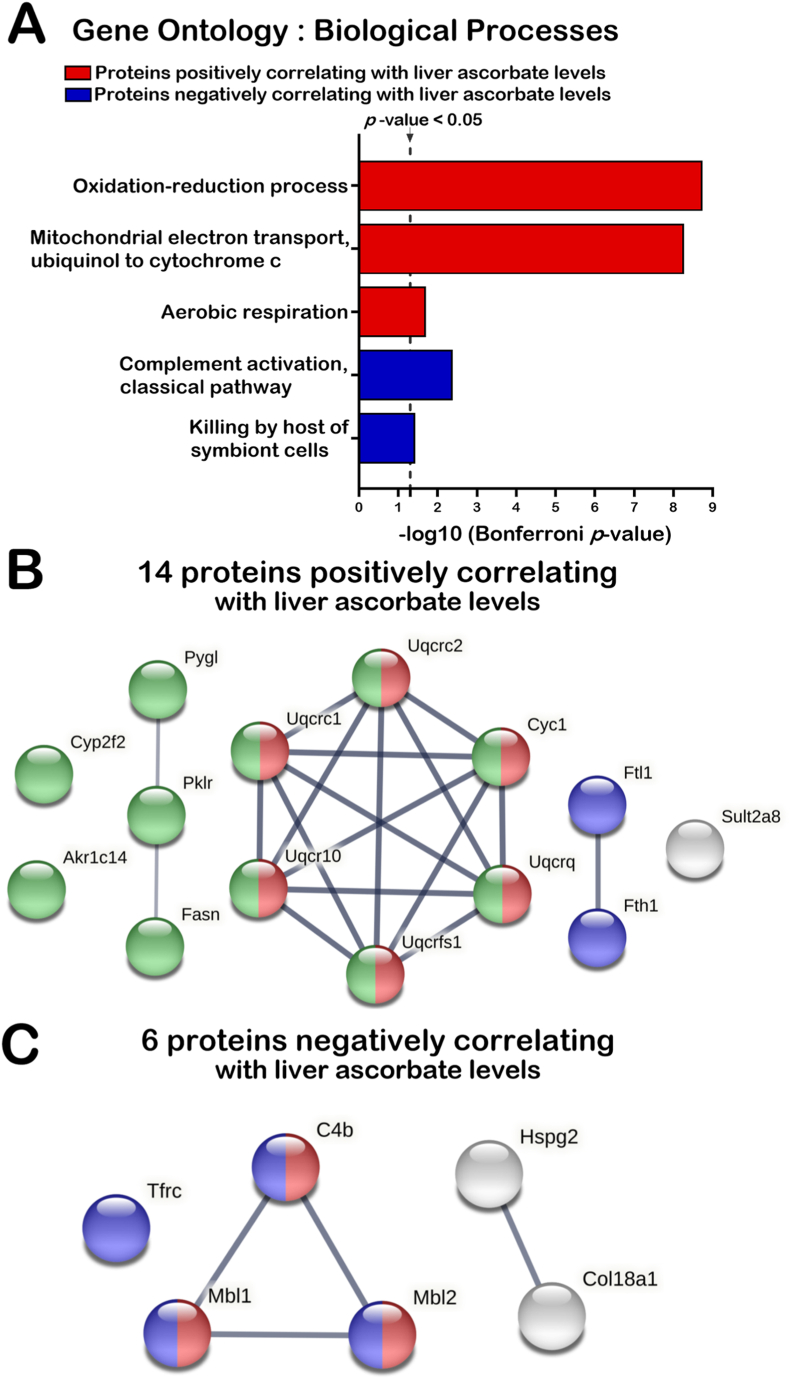
Gene Ontology and protein network analyses showing the biological processes correlating with liver ascorbate levels in both males and females. (A) Biological processes analyses of proteins that correlated with liver ascorbate levels in both female and male mice using DAVID. Biological processes with Bonferroni *p*-value < 0.05 are presented in the histogram. (B) Protein-protein interaction networks of common proteins in females and males correlating positively with liver ascorbate levels using STRING. Colored nodes: green = carbohydrates and fatty acids metabolisms (FDR = 1.52 × 10^−7^); red = mitochondrial complex III of the electron transport chain (FDR = 7.29 × 10^–13^); blue = iron homeostasis (FDR = 0.0343); gray = hepatic sulfotransferase involved in the maintenance of bile acid homeostasis. (C) Protein-protein interaction networks of common proteins in female and males correlating negatively with liver ascorbate levels using STRING. Colored nodes: red = classical complement activation pathway (FDR = 0.0010); blue = positive regulation of immune response (FDR = 0.0055); gray = associated with the extracellular matrix. The protein nomenclatures are depicted in the supporting information Table 6. The thick edges represent physical interactions. The thin edge represents co-expression in the same metabolic pathway. (For interpretation of the references to colour in this figure legend, the reader is referred to the Web version of this article.)

We next investigated whether the proteins that commonly correlated with liver ascorbate levels in both females and males were associated to specific biological processes. For the 14 common proteins positively correlating with liver ascorbate levels, we found a significant enrichment for proteins involved in oxidation-reduction process, the electron transport from ubiquinol to cytochrome *c* in mitochondria, and in aerobic respiration (Bonferroni *p*-value < 0.05) (Fig. 6A). To further highlight the functional links between the 14 proteins that positively correlated with liver ascorbate levels, we used STRING (Search Tool for the Retrieval of Interacting Genes), which is a meta-database program that generates a network of protein interactions from high-throughput experimental data, literature, and predictions based on genomic context analysis [26]. Interestingly, six of the 14 proteins were functionally linked as they are part of the mitochondrial complex III of the electron transport chain and include Uqcrc1, Uqcrc2, Uqcrc10, Uqcrq, Uqcrfs1 and Cyc1 (highlighted in red on Fig. 6B). Fig. 6B also indicates that Pygl, Pklr, and Fasn are functionally linked to carbohydrates and fatty acids metabolisms. With Cyp2f2 and Akr1c14, all these proteins are involved in metabolism processes as highlighted in green in Fig. 6B based on our STRING analysis. Finally, among the 14 proteins that positively correlated with liver ascorbate levels, Ftl1 and Fth1 proteins were both functionally linked to iron homeostasis and play a role in delivery of iron to cells (highlighted in blue in Fig. 6B).

DAVID analysis on the six proteins that inversely correlated with liver ascorbate levels in both females and males revealed several proteins involved in the classical complement activation and the killing by host of symbiont cells (Bonferroni *p*-value < 0.05) (Fig. 6A). Similarly, STRING analysis indicated that three proteins (C4b, Mbl1, and Mbl2) were functionally linked to the classical complement activation pathway (highlighted in red on Fig. 6C). Furthermore, the C4b, Mbl1, and Mbl2 are implicated in the positive regulation of immune response with Tfrc (highlighted in blue in Fig. 6C). Finally, Col18a1 and Hspg2 were functionally associated with the extracellular matrix based our STRING analysis (highlighted in gray in Fig. 6C).

### 3.7 Validation of the differential expression of mitochondrial complex III proteins by immunoblot analyses in ascorbate deficient *Gulo^−/−^* mice

**Fig. 7:**
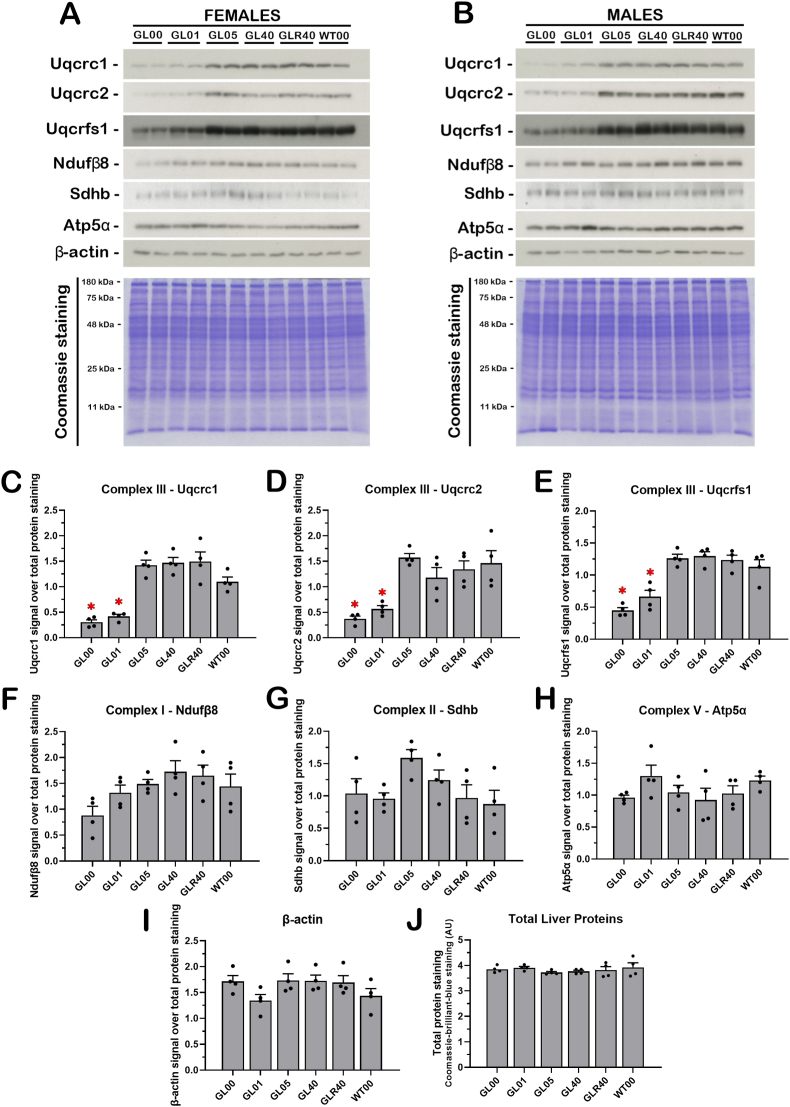
Immunoblot analyses in ascorbate deficient *Gulo^−/−^* mice livers validating the differential expression of mitochondrial complex III proteins. Analyses of six different proteins from the mitochondrial electron transfer chain complexes by western blots on whole liver lysates for females (A) or males (B). Signal quantification of three proteins from the complex III over total protein staining: Uqcrc1 (C), Uqcrc2 (D), Uqcrfs1 (E). (F) Ratio between the Ndufβ8 protein signal from the complex I and total protein staining. (G) Ratio between the Sdhb protein signal from the complex II and total protein staining. (H) Ratio between the Atp5α protein signal from the complex V and total protein staining. (I) Ratio between the β-actin protein signal and total protein staining. (J) Coomassie-brilliant-blue staining quantification. Bars in each histogram represent mean ± SEM (N = 4 females and males combined per group). Significant differences are indicated using * (with a Tukey post ANOVA tests *p*-value < 0.05). (For interpretation of the references to colour in this figure legend, the reader is referred to the Web version of this article.)

Three proteins of the mitochondrial complex III were further analyzed by western blots on total lysate from the liver of all our groups of mice (Fig. 7A for females and 7B for males). As indicated in Fig. 7, the complex III proteins Uqcrc1, Uqcrc2, and Uqcrfs1 showed significantly lower levels in the GL00 and GL01 groups compared to the GL05, GL40 and WT groups in both females and males (Fig. 7A–E; ANOVA *p*-values < 0.001). In contrast the proteins Ndufβ8, Sdhb, and Atp5α, which are part of the mitochondrial complex I, II, and V of the mitochondrial electron transfer chain, respectively, did not exhibit significant level changes between the different mouse cohorts (Fig. 7F–H). Similarly, the level of β-actin did not significantly change between the different mouse groups (Fig. 7I). Total liver proteins revealed by Coomassie staining were similar in all the groups (Fig. 7J). These immunoblot results confirm the lower expression of several complex III proteins in *Gulo*^*−/−*^ mice treated with 0 and 0.01% of ascorbate compared to the other groups of *Gulo*^*−/−*^ mice treated with higher levels of ascorbate in drinking water that we identified in our LFQ analyses.

### 3.8 *Gulo*^*−/−*^ mice treated with suboptimal levels of ascorbate in drinking water exhibit lower complex III activity, lower ATP levels, and increased ROS in total liver

**Fig. 8:**
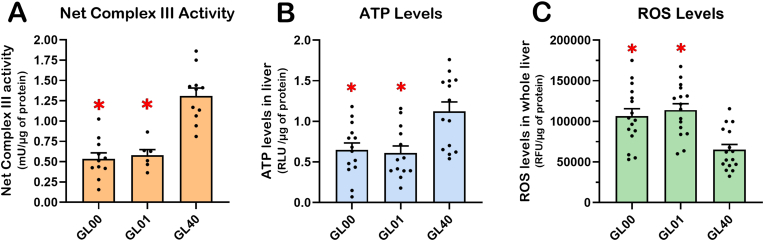
Suboptimal concentrations of vitamin C impact on mitochondrial complex III activity, ATP, and ROS levels in *Gulo*^*−/−*^ mice liver. (A) Mitochondrial complex III activity measurements in MEE samples from female and male GL00, GL01, and GL40 mice groups (N = 6–11 females and males combined per group, ANOVA *p*-values < 0.0001). (B) ATP levels quantification in whole liver lysates of female and male GL00, GL01, and GL40 mice (N = 13–14 females and males combined per group, ANOVA *p*-values < 0.001). (C) ROS levels evaluation using whole liver lysates of female and male GL00, GL01, and GL40 (N = 16 females and males combined per group, ANOVA *p*-values < 0.0001). Bars in each histogram represent mean ± SEM. Significant differences are indicated using * (with a Tukey post ANOVA tests *p*-value < 0.05).

Since the levels of six of the mitochondrial complex III proteins were lower in the *Gulo*^*−/−*^ mice treated with 0 and 0.01% of ascorbate (GL00 and GL01) compared to *Gulo*^*−/−*^ mice treated with 0.4% ascorbate (GL40) in drinking water based on the LFQ analysis, we measured the activity of the complex III in the MEE samples. As indicated in Fig. 8A, the activity of mitochondrial complex III was ∼2.5-fold lower in the GL00 and GL01 mice than in the GL40 mice (ANOVA *p*-value < 0.001 for males and females combined).

To determine whether the lower mitochondrial complex III activity had an impact on ATP levels in the whole liver of *Gulo*^*−/−*^ mice treated with suboptimal levels of ascorbate in drinking water, ATP was measured in the GL00, GL01, and GL40 groups (males and females combined). As indicated in Fig. 8B, the ATP levels (per μg of total liver proteins) were significantly lower in the GL00 and GL01 groups than in the GL40 group of mice.

Finally, we determined whether ROS was increased in the whole liver of *Gulo*^*−/−*^ mice (males and females combined) treated with suboptimal levels of ascorbate in drinking water. As indicated in Fig. 8C, there was a significant increase in ROS levels determined by the fluorogenic dye DCFA in the GL00 and GL01 groups compared to the GL40 group of mice.

## 4 Discussion

Several studies with *Gulo*^*−/−*^ mice have reported the consequence of vitamin C insufficiency in induced liver injury and point to a hepatoprotective effect of this vitamin in vivo [[27], [28], [29]]. Additional reports have indicated that even before inducing liver injury, vitamin C deficient *Gulo*^*−/−*^ mice exhibit metabolic alterations [30] that impacts liver functions [20,31]. More recently, it has been shown that the levels of several proteins secreted in the blood by the liver correlate positively or inversely with the serum ascorbate concentrations in *Gulo*^*−/−*^ mice [21]. Such proteins are involved in platelet activation response, extracellular matrix−receptor interactions, focal adhesion, carbon metabolism, and complement and coagulation cascades. However, the connection between these biological pathways and the endoplasmic reticulum stresses or the mitochondrial dysfunctions observed in the liver of ascorbate-deficient *Gulo*^*−/−*^ mice [20] is unclear. To gain more insight into the biochemical pathways altered in the liver of *Gulo*^*−/−*^ mice treated with different amounts of ascorbate in drinking water, we used a LFQ mass spectrometry strategy [32] on hepatic microsomal enriched extracts (MEE). We obtained proteins of the MEE fraction by differential centrifugations. The supernatant from the final centrifugation contained proteins that were not only part of the cytosol like Hsc70, but also from disrupted cellular surface membrane that occurred during the first steps of the process as seen by the strong signal of Svct1in the western blots (Fig. 2B). Although some Svct1 membrane proteins were also found in the MEE fraction, the use of this enrichment method allowed the identification of 3276 proteins that were mostly classified as mitochondrial, endoplasmic reticulum, and intracellular vesicles (Fig. 2C). Importantly, the levels of hepatic ascorbate did not impact on the efficiency of the extraction procedure in our different mouse groups (Fig. 2B).

The levels of ascorbate in the hepatic tissue of our different mouse groups were consistent with the levels of serum ascorbate that we previously measured in these mice [20] with high significant Pearson correlation values in both males and females (Fig. S1). Furthermore, *Gulo*^*−/−*^ females treated with 0.4% ascorbate in drinking water exhibited higher levels of hepatic ascorbate concentrations compared to *Gulo*^*−/−*^ males (Fig. 1D), which is consistent with what we have observed in the serum of these mice [20]. Cells can accumulate ascorbate through the sodium-dependent vitamin C transporters encoded by the two genes *Slc23a1* and *Slc23a2* in rodents [33]. It has been reported that female mice have higher ascorbate concentrations in plasma and in liver, a tissue that expresses predominantly Slc23a1 [34]. Furthermore, knockout mice of the ascorbate transporter Slc23a1 revealed differences in urinary ascorbate excretion between males and females. The impact of *Slc23a1* knockout was much greater on ascorbate renal reabsorption in females than in male mice [35]. The higher levels of serum and liver ascorbate levels in females compared to males are consistent with such findings. In addition to the ascorbate concentration differences between male and female hepatic tissues, principal component analyses of the LFQ data indicated that the proteome profiles of *Gulo*^*−/−*^ mice were different between the MEE of males and females in all the various ascorbate treatments (Fig. 3B). The reason for the differences between male and female mice is unknown but may be caused by sexual hormone dimorphism [[36], [37], [38]].

We determined whether we could rescue the liver proteome profile of ascorbate-deficient *Gulo^−/−^* mice by simply adding back ascorbate in drinking water to re-establish hepatic ascorbate concentrations to WT levels. The supplementation of ascorbate-deficient *Gulo^−/−^* males with 0.4% ascorbate in drinking water for four weeks not only normalized hepatic ascorbate to WT levels (Fig. 1C), but also established a liver proteome profile that did not significantly differ from *Gulo^−/−^* males that never experienced ascorbate deficiency during their lifetime based on our PCA (Fig. 4B). In contrast, treating ascorbate-deficient *Gulo^−/−^* females with 0.4% ascorbate in drinking water for four weeks did not re-establish the proteome profile observed in *Gulo^−/−^* females that never experienced ascorbate deficiency during their lifetime (Fig. 4A), even though hepatic ascorbate levels were the same in both groups (Fig. 1B). These results are consistent with the greater number of MEE proteins, which levels were significantly altered by ascorbate treatments in our different groups of females compared to males (Fig. 4C). These findings are also consistent with the greater number of serum proteins altered in the serum of *Gulo^−/−^* females compared to males treated with different concentrations of ascorbate in drinking water [39]. Note that ascorbate-deficient *Gulo^−/−^* mice were treated only four weeks. It is possible that a longer treatment period with 0.4% ascorbate in drinking water may be required to completely re-establish the MEE proteome profile in females. This will require different timepoints of ascorbate induced deficiency in our mouse cohorts as it has been reported that even small age differences can lead to significant different plasma metabolomes in mice impairing reproducibility [40]. Regardless of the time frame that would be required to completely rescue the hepatic proteome profile in *Gulo^−/−^* females, the Pearson correlation analyses indicated that the response to various ascorbate concentrations in drinking water differed between *Gulo^−/−^* females and males. Gene ontology analysis on the LFQ intensities of the identified proteins that correlated significantly and positively with hepatic ascorbate levels in female mice included mitochondrial electron transport, epoxygenase P450 pathway, steroid, lipid, xenobiotic, and organic acid metabolic processes (Table 2). The proteins that correlated inversely with liver ascorbate levels are involved in RNA splicing, mRNA processing, and the innate immune response. The males showed only a significant positive correlation between proteins associated with the mitochondrial electron transport and liver ascorbate levels (Table 2). These results indicate that more biochemical pathways are affected in the liver MEE by varying the amount of ascorbate in the diet of female mice than in male mice. Noticeably, it has been reported that ascorbate deficiency in *Gulo^−/−^* females expressing the human lipoprotein(a) not only exhibit a less healthy metabolic lipid profile, but also show impaired estrogen secretion [41]. Consistent with these observations, it has been reported that oral ascorbate intake increases plasma estradiol in postmenopausal women specifically exhibiting low basal levels plasma vitamin C [42]. Furthermore, dehydroascorbate taken up by glucose transporters (which is subsequently reduced to ascorbate intracellularly) stimulates estradiol production by inducing several steroidogenic enzymes [43]. Estrogens in return are known to increase gene expression and elicit the epoxygenase P450 pathway, steroid and lipid metabolic processes as well as several xenobiotic metabolic processes more efficiently in females than in males in different tissues [[44], [45], [46], [47], [48], [49]].

Although *Gulo^−/−^* females and males exhibited differences in the number of proteins that correlated with liver ascorbate levels, fourteen and six proteins correlated positively and inversely with hepatic ascorbate levels, respectively, in both *Gulo^−/−^* females and males (Fig. 5, Fig. 6). Various proteins of the complement activation pathway inversely correlated with hepatic ascorbate levels in both males and females. This result is consistent with a study reporting an inverse correlation between plasma ascorbate levels and several components of the complement activation pathway (including C4b) in a human cohort [50]. The complement system is an essential element of the innate immune response that becomes activated upon recognition of molecular patterns associated not only with pathogens, but also with abnormal cells and modified molecules in the extracellular environment [51]. The abnormal mitochondrial morphology [20], the increase of ROS in the liver, and the altered secretion of serum proteins (including proteases) in ascorbate-deficient *Gulo^−/−^* mice [21] are likely sources of complement activation.

STRING analysis also revealed that the most significant assembles affected by ascorbate deficiency included proteins involved in carbohydrates and fatty acids metabolisms as well as proteins of the mitochondrial complex III in the electron transport chain (Fig. 6B). Interestingly, the LFQ analysis did not reveal correlation of proteins of the other mitochondrial electron transport chain complexes with the levels of hepatic ascorbate levels. Complex III of the electron transport chain is a symmetrical dimer composed of 10 subunits per monomer. It transfers electron from ubiquinol to cytochrome *c* [52,53]. Nine subunits are encoded by nuclear genes [53]. Eight of these subunits were identified in the LFQ analysis. Six of the complex III subunits showed a positive correlation with hepatic ascorbate levels based on our LFQ analysis in both females and males. Although, the Western blot analyses on three of these complex III proteins (Uqcrc1, Uqcrc2, and Uqcrfs1) in whole liver lysates did not show a gradual increase with the hepatic levels of ascorbate, the immunoblot results undoubtedly confirmed the lower expression of these complex III proteins in *Gulo*^*−/−*^ mice treated with 0 and 0.01% of ascorbate compared to the other groups of *Gulo*^*−/−*^ mice treated with higher levels of ascorbate (Fig. 7). The Western blot analyses also demonstrated that the levels of proteins from complex I (Ndufβ8), complex II (Sdhb), and complex V (Atp5α) were not significantly different between *Gulo^−/−^* females and males treated with suboptimal or optimal levels of ascorbate (Fig. 7) as found in the LFQ analysis. Thus, the Pearson correlation analyses between the intensities of the LFQ and the hepatic ascorbate levels were stringent enough in the present study (Pearson coefficient *r* > |0.5897| and a *p*-value < 0.01 for N = 18) to discern the proteins that were significantly lower in *Gulo^−/−^* mice exhibiting suboptimal levels of ascorbate (less than 50 ng of ascorbate per mg of total liver protein in Fig. 1) in the Western blot analyses. More importantly, complex III activity was significantly decreased in *Gulo^−/−^* mice treated with suboptimal ascorbate concentrations (0% and 0.01%) compared to mice treated with optimal levels (0.4%) of ascorbate (Fig. 8A). Concomitantly, ascorbate-deficient *Gulo^−/−^* mice exhibited decreased ATP levels in their whole liver tissue (Fig. 8B). It is known that an inhibition of the mitochondrial complex III with either antimycin A or myxothiazol significantly decrease ATP levels [54]. Thus, a decrease in ATP in the whole liver is likely a consequence of the decreased complex III activity observed in ascorbate-deficient *Gulo^−/−^* mice.

Ascorbic acid, the main form of vitamin C in cells, functions as a reductant during the biosynthesis of collagen, carnitine and catecholamine thereby producing dehydroascorbic acid (DHA) [55]. In mitochondria local gradients might occur with a transient increase of DHA in regions of high ROS production at the respiratory chain, where ascorbic acid acts as a major antioxidant. Furthermore, ascorbic acid transport into mitochondria takes place in form of DHA via the glucose transporter GLUT1 [56]. Note that, once ascorbate has exerted its antioxidant effect and has been fully oxidized to DHA, it must be recycled or will be irrevocably lost within several minutes at physiologic pH due to ring-opening of DHA. Using specific inhibitors of the mitochondrial electron transport chain, it was found that the site of ascorbate recycling is localized to a portion of complex III with exposure to the outer surface of the inner mitochondrial membrane [57]. Thus, by affecting mitochondrial complex III protein levels and activity, the suboptimal levels of ascorbate in the liver would also alter its maintenance in the mitochondria [58] and hence potentially lead to the increase ROS observed in the whole liver of *Gulo^−/−^* mice (Fig. 8C).

Hypovitaminosis C is becoming a pressing issue in the elderly and in chronically ill hospitalized patients [[7], [8], [9], [10]]. Although an increase in inflammation and oxidative stress status were identified in these patients, there was no information on the activity of the mitochondrial electronic transfer chain in such subjects. Normal human fibroblasts are known to exhibit increased oxidative stress and decreased mitochondrial enzymatic respiratory chain activities with the number of passages in culture. A few studies have reported that increasing the concentration of ascorbate in such aged cultured human fibroblasts increased their mitochondrial respiratory chain activities including complex III function [59,60]. However, although complex III protein levels were not examined in such studies, the data on the increased mitochondrial respiratory chain activities may suggest that ascorbate is not only acting as an antioxidant but may also regulate the levels of specific mitochondrial subunits in human cells. An in-depth analysis of the impact of ascorbate on complex III protein levels in various tissues in human patients with hypovitaminosis C or with different degenerative diseases will be required to unveil the mechanisms linking vitamin C to mitochondrial subunit regulation in humans.

In conclusion, the present mass spectrometry study indicates that an ascorbate-deficiency in mice decreases the levels of several proteins that are part of the mitochondrial complex III in the liver. These findings provide new information on how ascorbate deficiency potentially induces mitochondrial dysfunction in the liver of mice. Thorough analyses of the transcriptome and translatome in this mouse model are underway to understand how ascorbate specifically modulates the levels of these proteins in vivo.

## Author contributions

L.A. and M.L. conceived the project and processed the liver samples. S.B. and C.G. performed the proteomic experiments and analyzed the data. A.D. supervised the mass spectrometry experiments. L.A., S.B., C.G., and M.L. wrote the manuscript.

## Declaration of competing interest

The authors declare that they have no known competing financial interests or personal relationships that could have appeared to influence the work reported in this paper.

## Data Availability

No data was used for the research described in the article.
